# Assembling Surfactants-Mucoadhesive Polymer Nanomicelles (ASMP-Nano) for Ocular Delivery of Cyclosporine-A

**DOI:** 10.3390/pharmaceutics12030253

**Published:** 2020-03-11

**Authors:** Eleonora Terreni, Patrizia Chetoni, Silvia Tampucci, Susi Burgalassi, Ali Athab Al-kinani, Raid G. Alany, Daniela Monti

**Affiliations:** 1 Department of Pharmacy, University of Pisa, 56126 Pisa, Italy; eleonora.terreni@farm.unipi.it (E.T.); patrizia.chetoni@unipi.it (P.C.); silvia.tampucci@unipi.it (S.T.); susi.burgalassi@unipi.it (S.B.); 2 Inter-University Center for the Promotion of the 3Rs Principles in Teaching & Research (Centro 3R), 56126 Pisa, Italy; 3 Drug Discovery, Delivery and Patient Care (DDDPC) Theme, School of Life Sciences, Pharmacy and Chemistry, Kingston University London, Kingston upon Thames, London KT1 2EE, UK; a.alkinani@kingston.ac.uk (A.A.A.-k.); R.Alany@kingston.ac.uk (R.G.A.); 4 School of Pharmacy, The University of Auckland, Auckland 1010, New Zealand

**Keywords:** nanomicelles, ocular pharmacokinetic, rabbits, hyaluronic acid, vitamin E-TPGS, cyclosporine A, reconstituted corneal tissue

## Abstract

The physiological protective mechanisms of the eye reduce the bioavailability of topically administered drugs above all for those with high molecular weight and /or lipophilic characteristics, such as Cyclosporine A (CyA). The combined strategy based on the association of nanomicelles and mucoadhesive polymer seems promising since a limited number of commercial products containing CyA have been recently approved. The scope of this investigation was the design of Assembling Surfactants-Mucoadhesive Polymer Nanomicelles (ASMP-Nano), based on a binary system of two surfactants in combination with hyaluronic acid, and their biopharmaceutical evaluation. The optimisation of the ASMP-Nano in term of the amount of surfactants, CyA-loading and size determined the selection of the clear and stable Nano1HAB-CyA formulation containing 0.105% *w*/*w* CyA loaded-nanomicelles with a size of 14.41 nm. The nanostructured system had a protective effect towards epithelial corneal cells with a cell viability of more than 80%. It interacted with cellular barriers favouring the uptake and the accumulation of CyA into the cells as evidenced by fluorescent probe distribution, by hindering CyA permeation through reconstituted corneal epithelial tissue. In pharmacokinetics study on rabbits, the nanomicellar carrier prolonged the CyA retention time in the precorneal area mainly in presence of hyaluronic acid (HA), a mucoadhesive polymer.

## 1. Introduction

The eye possesses innumerable efficient protective and defensive mechanisms (e.g., corneal epithelium, blinking, tearing and tear film turnover, mucus) useful in removing or immobilising foreign substances or exogenous chemicals, such as drugs or nanocarriers, from the ocular surface, avoiding their corneal penetration [1,2]. 

Due to its physiological protective mechanisms the achievement of therapeutic levels of the drugs into ocular tissues became an extremely arduous challenge, especially for drugs with high molecular weight and/or hydrophobic characteristic, such as Cyclosporine A (CyA), a macrocyclic polypeptide having MW 1202 Da, log Po/w = 1.4–3.0 and a water solubility less than 0.1 mg/mL [3,4,5]. As early as 1980, when the first application of CyA as immunosuppressive agent for the treatment of corneal allograft rejection and ocular inflammation was studied, several efforts have aimed to develop CyA-based ocular delivery systems. The ocular application of CyA formulations remains preferred and advantageous to treat ophthalmic pathologies such as uveitis, corneal healing, vernal keratoconjunctivitis and dry eye disease [6,7,8], without causing the systemic effects associated with other routes of administration (oral, parenteral etc.) for the treatment of ocular pathologies [9,10,11]. CyA is difficult to formulate in aqueous solution at a therapeutic concentration. 

Over the past two decades, research in the ophthalmic field has produced a limited number of commercial formulations based on Cyclosporine-A useful for the treatment of dry eye disease (DED), mainly consisting of coarse or nano- emulsions. Restasis® from Allergan (Irvine, CA, USA) is a 0.05% CyA anionic emulsion, containing castor oil and polysorbate 80 as emulsifying agent, and was launched on the United States market before 2012, while Ikervis® (Santen Oy, Tampere, Finland), a 0.1% CyA stable cationic nanoemulsion based on Nanosorb® technology, was granted marketing authorisation by the European Commission (EC) for treatment of severe keratitis in adult patients with DED only after 2015. These commercial products result from many attempts done by using different strategies, still in continuous development, such as prodrugs [12], liposomes [13], nanoparticles [14], nanosphere [15], solid lipid nanoparticles [16,17,18], contact lenses [19], ocular inserts [20,21], implants [22] and emulgel [23]. Each of these techniques has contributed to solve various critical issues such as cytotoxicity, low precorneal residence time after ocular instillation with reduced bioavailability and low solubility. 

Furthermore, several authors reported studies on micellar solubilisation of CyA by non-ionic surfactants that tends to form clustered aggregates (micelles) at/or above their critical micelle concentration (CMC). Micellization generally occurs when the intermolecular forces, such as hydrophobic, steric, electrostatic, hydrogen bonding and Van der Waals forces are balanced. Other factors that affect the assembling of micelles include the shape, charge and size of surfactant monomers, ionic strength, pH, temperature, total surfactant concentration and the composition of the surfactants’ mixtures [24,25]. The nanomicelles favour solubilisation processes by sequestering the drug in the lipophilic core, formed by the non-polar tails of the surfactant, and interacting with the hydrophilic environment through an outer shell consisting of polar or charged heads. Several reports support the use of surfactant micelles for improved penetration of topically applied drugs through the cornea and enhanced ocular bioavailability [26,27,28,29,30]. Recently (2018), the FDA approved the first commercial product containing 0.09% CyA based on micellization technology (Cequa from Sun Pharmaceutical Ind., Cranbury, NJ, USA) [31]. 

The scope of the present investigation was the design of Assembling Surfactants-mucoadhesive Polymer Nanomicelles (ASMP-Nano) based on a binary system of two surfactants (d-α-Tocopherol polyethylene glycol succinate, VitE-TPGS, and octylphenoxy poly(ethyleneoxy)ethanol, OPEE) in combination with hyaluronic acid (HA) for the ocular delivery of CyA. The mixture of surfactants allowed to obtain nanomicelles able to solubilise an active pharmacologically CyA amount by using the surfactants concentration as minimal as possible [32]. VitE-TPGS is a well-known, safe water-soluble derivative of vitamin E. This non-ionic surfactant was used in blend with OPEE that belongs to the series of ethoxylated octylphenol, both with similar HLB values (HLB index of about 13) and with low CMC. 

They are used in many commercial products due to their biocompatibility and minimal toxicity compared with other cationic, anionic or amphoteric polymeric surfactants, even if literature data report about the minimal hemolytic activity, toxicity, irritation and inflammation to the ocular structures. They appear to meet the requirements of ocular delivery and seem a good starting point to be optimised with the addition of HA. The well-known mucoadhesive properties of HA could favour the interaction with the mucus of corneal surface improving the CyA ocular bioavailability and could also protect the corneal epithelium from possible damage caused by the surfactants that form the nanomicelles.

The optimisation of the ASMP-Nano formulation in term of CyA-loading into nanomicellar lipophilic core and of promotion of the ASMP-Nano ocular residence time is a promising field of investigation. The development of ophthalmic formulations containing hyaluronic acid has been pursued for many years, but its influence on the behaviour of nanomicellar systems in term of capability to interact with the ocular barriers and increase the ocular residence time has not yet been investigated.

## 2. Materials and Methods

### 2.1. Materials

#### 2.1.1. Chemicals

The following materials were used: pharmaceutical grade d-α-Tocopherol polyethylene glycol succinate, free sample from BASF (VitE-TPGS, Kolliphor^®^TPGS, BASF, Ludwigshafen, Germany); octylphenoxy poly(ethyleneoxy)ethanol (OPEE, Igepal® CA-630 viscous liquid, Sigma-Aldrich, Milan, Italy); pharmaceutical grade hyaluronic acid (HA, 1,65 MDa, Chemofin s.r.l, Milan, Italy); European Pharmacopoeia grade cyclosporine A (CyA), kind gift from Poli Industria Chimica S.p.A (Milan, Italy); Fluorescein Isothiocyanate (FITC, Isomer I, 90%, ACROS Organics™, Geel, Belgium); WST-1 cell proliferation (cat no. 1644807, Roche Diagnostics GmbH, Germany). 

All other chemicals and solvents were of analytical grade. Ultrapure water was prepared using Milli-Q® plus apparatus (Millipore, Milan, Italy).

#### 2.1.2. Cell Cultures and Animals

The rabbit corneal epithelial cell line (RCE, European Cell Culture Collection (N° 95081046, ECACC, Salisbury, UK) was used for cytotoxicity test. The growth medium had the following composition: Dulbecco’s modified Eagle’s medium (DMEM) with Ham’s nutrient mixture F12 (1:1) added of L-glutamine (1% *v*/*v*), penicillin (100 IU/mL), streptomycin (0.1 mg/mL), amphotericin B (0.25 µg/mL), foetal bovine serum (15% *v*/*v*) (Gibco Invitrogen S.r.l., Milan, Italy), epidermal growth factor (10 ng/mL) and insulin (5 mg/mL) (Sigma Chemical Co., St Louis, MO, USA).

COR-100 EpiCorneal^TM^ reconstituted tissues were obtained from MatTek Corporation (Ashland, MA, USA) and were used following the experimental procedure proposed by MatTek.

Male New Zealand albino rabbits weighing 2.8–3.5 kg were purchased from Pampaloni Rabbitry (Pisa, Italy). They were housed in standard cages in a light-controlled room (10 h dark/14 h light cycle) at 19 ± 1°C and 50 ± 5% relative humidity and were given a standard pellet diet and water ad libitum. During the experiments the rabbits were placed in restraining boxes to which they had been habituated in a room with dim lighting and were allowed to move their heads and eyes freely [33]. For in vivo studies the rabbits were used and treated according to the “Guide for the Care and Use of Laboratory Animals”. All experimental procedures were carried out in accordance with the ARVO Statement for the Use of Animals in Ophthalmic and Vision Research and the European Union guidelines for the use of animals in research, and were approved by the Ethical and Scientific Committee of University of Pisa and carried out under veterinary supervision (Authorisation n. 350/2018-PR, 11/05/2018).

### 2.2. Methods 

#### 2.2.1. Preparation of Assembling Surfactants-Mucoadhesive Polymers Nanomicelles (ASMP-Nano) 

Assembling Surfactants-Mucoadhesive Polymers Nanomicelles (ASMP-Nano) containing Cyclosporine-A based on a binary mixtures of two non-ionic surfactants (X) and hyaluronic acid (HA_y_) chosen as mucoadhesive polymer (NanoXHA_y_-CyA), were prepared by adding an accurately weighted amount of CyA to different percentage of VitE-TPGS and OPEE mixture (2.25:1.0 weight ratio) in the range of 1.5 to 0.5% *w*/*w*. In detail, VitE-TPGS was melted at 50 °C in a closed vial for one hour; then, OPEE and CyA (0.15% *w*/*w*) were added and mixed together to obtain a homogeneous blend. Secondly, an HA aqueous dispersion in isotonic phosphate buffer solution (PBS, pH = 7.4, 66.7 mM) were added at the same blend temperature. The final mixture was then stirred overnight and filtered through sterile filters (0.2 μm RC Syringe filter, Phenomenex^®^, Torrance, CA, USA) to remove unloaded drug, aggregates and other foreign particulates.

Three different percentages of total surfactants mixture (1.5, 1.0 and 0.5% *w*/*w*) were selected and, for each one, three different concentration of HA (0.001, 0.01 and 0.1% *w*/*w*) was used.

In addition, two different reference nanomicellar formulations were prepared by applying the same experimental procedure and based on the same couple of surfactants at 1.0% *w*/*w* concentration, but the first contained CyA (0.15% *w*/*w*) without HA (Nano1-CyA), and the second contained HA (0.01% *w*/*w*) but without CyA (Nano1HA_B_). The composition of all prepared mixtures of ASMP-Nanomicellar dispersions are reported in Table 1.

**Scheme 1:**
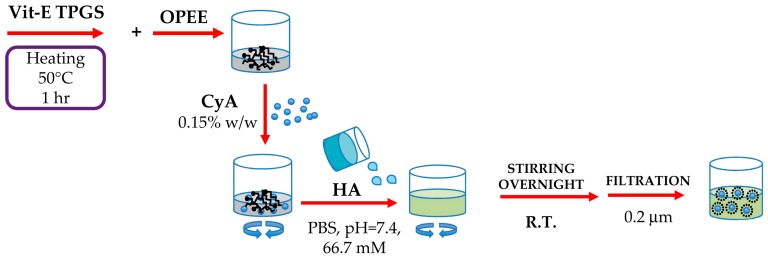
Schematic representation of the different steps involved in the preparation of ASMP-Nano.

The osmolality and pH values of the formulations were determined by a freezing point depression method using an Osmometer (5R version, Roebling, Germany) and a digital pH-meter (611 model, Orion Research, USA), respectively. A schematic representation of the nanomicelles preparation process is summarised in Scheme 1.

##### Design of Experiment (DOE) Optimisation Study

A suitable Design of Experiment (DOE) study was planned to screen the influence of the percentages of the surfactants mixture and amount of the mucoadhesive polymer (HA) on three selected dependent variable amounts of solubilised CyA, CMC value and size of nanomicelles. A full-factorial design with 3 × 3 quadrature points in the two independent variables was chosen and the three quantities were experimental measured for each of the nine binary mixtures (see Table 1, mixtures 1–9). A second-order-polynomial response surface was computed by using the software MATLAB, MatWorks^®^ for the selected three output quantities, namely solubilised CyA (Y1, CyA-In), hydrodynamic diameter (Y2, D_h_) and Critical Micelle Concentration value (Y3, CMC), as a function of two independent variables: X1 = total percentage of used surfactants (Vit E-TPGS and OPEE 2.25:1 constant ratio) and X2 = percentage of HA.

#### 2.2.2. Preparation of Assembling Surfactants-Labeled Mucoadhesive Polymers Nanomicelles (ASLMP-Nano)

ASLMP-Nano was prepared by using a fluorescein-labelled hyaluronic acid (HA-FITC) that was appropriately synthetized following already reported procedures [34,35]. Sodium hyaluronate (HA, 0.5 g) was dissolved in dimethyl sulfoxide (DMSO, 5.0 mL) containing 50 μL of pyridine at 200 °C to facilitate solubilisation. Then, fluorescein isothiocyanate (0.05 g) and subsequently dibutyltin dilaurate (10 μL) were added, and the obtained mixture was heated for two hours at 95 °C under continuous stirring. The HA-FITC derivative was first precipitated by ethanol and then separated by filtration washing the residue several times with the same solvent to remove the excess of unreacted dye. The final product was dried under vacuum and stored in a desiccator. The aqueous dispersion of the labelled mucoadhesive polymer (HA-FITC) showed evident signs of fluorescence under a UV-lamp at λ = 366 nm.

The fluorescent derivative HA-FITC was analysed by differential scanning calorimetry (DSC) and attenuated total reflectance Fourier transform infrared spectroscopy ATR-FITR and was used to prepare the ASLMP-Nano formulation (Nano1HA_B_FITC-CyA) following the method described in Section 2.1, and during the whole preparation process the polymeric dispersion was maintained protected from the light. 

#### 2.2.3. Physicochemical Characterisation 

##### Dynamic Light Scattering Analysis

The average hydrodynamic diameter (D_h_) and polydispersity index (PDI) of all ASMP-Nano formulations were determined by measuring the rate of fluctuations in laser light intensity scattered by the nanomicelles immediately after their preparations using the Dynamic Light Scattering (DLS) analysis (Beckman Coulter® N4 Plus, Beckman Coulter s.r.l., Milan, Italy). Five minutes prior to light-scattering measurements each sample was diluted with ultrapure water previously filtered through 0.45 μm pore size filter (polyethersulfone membrane, Millipore Express PLUS, Merck, Italy). The final concentration was chosen on the basis of the measurement intensity ranging from 5 × 10^4^ to 1 × 10^6^ counts per second (cps). The average size for each ASMP-Nano formulation was obtained on three different samples for which six runs were carried out using an angle of 90° [30] and run time of 200 s at 20 °C. 

The same protocol analysis was performed to characterise the Nano1HA_B_FITC-CyA formulation.

##### Determination of Critical Micellar Concentration of ASMP-Nano Mixtures

Critical micellar concentration (CMC) was determined applying the partially modified method proposed by Cholkar et al. [36], with iodine as probe. 

Briefly, the nanomicelles dispersions based on the different proportion of VitE-TPGS/OPEE/HA (see Table 1) in absence of CyA were prepared following already reported experimental method (paragraph 2.2). The mixtures were diluted with ultrapure water to obtain a series of preparations with a concentration of surfactants ranging between 0.001 and 0.2% *w*/*w*.

For the test, 25 μL of an iodine solution containing tri-iodide ion (I_3_**^-^**, 40.0 mM KI_3_), prepared by dissolving the iodine and potassium iodide (weight ratio 0.5:1.0) in ultrapure water, was added to an exactly measured volume (5.0 mL) of each dilution of the nanomicellar preparations. The resulting solutions were incubated in closed vials at room temperature for 15 h under dark and then the absorbance was measured at 366 nm to detect the hydrophobic I_2_ entrapped in the core of the nanomicelles. The test was repeated for each diluted sample in triplicate.

##### Determination of the Amount of Solubilised CyA (CyA-In) in the different ASMP-Nano Mixtures

The percentage of CyA solubilised by using the different mixtures of surfactants and mucoadhesive polymer was determined by RP-HPLC analysis after dilution of the nanomicellar formulations with organic solvent. An aliquot (1.0 mL) of each ASMP-Nano formulation (Table 1) was filtered through 0.2 μm pore size filter (Phenex RC Syringe filter, Phenomenex^®^, BO, Italy) and added with acetonitrile (ACN, 20.0 mL) to favour the dissolution of CyA into organic solvent. The amount of CyA was detected by HPLC analysis and each analysis was repeated on three different samples.

#### 2.2.4. Characterisation of the Selected ASMP-Nano Formulation (Nano1HA_B_-CyA)

##### Nanomicelles Yield (ASMP-Y), CyA Entrapment (CyA-EE) and CyA Loading Efficiency (CyA-LE) 

The prepared nanomicelles were characterised by yield, drug encapsulation efficiency and loading.

The nanomicelles yield was gravimetrically determined on a dried sample of Nano1HA_B_-CyA formulation according to Equation (1).

The experimental results on the amount of CyA solubilised by using the different surfactants mixtures, obtained according to paragraph 2.2.3, were utilised to calculate the percentage entrapment (CyA-EE) and loading efficiency (CyA-LD) of CyA according to Equations (2) and (3), respectively:

Nanomicelles Yeld (ASMP-Y) = (mass of nanomicelles ∗ 100)/(theoretical mass of total components)(1)

Percent of CyA entrapped (CyA-EE) = (mass of CyA in nanomicelles ∗ 100)/(mass of CyA added in the mixture)(2)

CyA loading efficiency (CyA-LE) = (mass of CyA in nanomicelles ∗ 100)/(mass of CyA added + mass of the other excipients)(3)

where the mass of CyA in nanomicelles corresponds to the amount of CyA solubilised (CyA-In).

##### Determination of Thermal Stability and Regeneration Time (RT) 

The stability of the selected nanomicellar formulation was primarily determined by visual observation and after an independent confirmation was obtained by turbidimetry. This analysis was performed both on the selected Nano1HA_B_-CyA formulation and on Nano1HA_B_ (reference). Briefly, aliquots (2.0 mL) of both nanomicellar preparations were placed into closed glass vials and heated up to 80 °C by using a water bath. The temperature was gradually increased by 10 °C every 5 min to allow the equilibration. The samples were observed and then their absorbance at 400 and 500 nm was measured (Spectrophotometer UV-2101PC, Shimadzu, Italy). The cloud point (CP) is defined as the temperature at which a solution containing non-ionic surfactants becomes turbid or milky-white and increases their absorbance [37]. The CP for ASMP-Nano formulations were determined at least in triplicate. 

Afterwards, the Nano1HA_B_-CyA and Nano1HA_B_ formulations were cooled down to room temperature and the Regeneration Time (RT), required to transform the cloudy dispersions into their original clear appearance was recorded. The Regeneration Time (RT), defined as the time required to attain the clarity observed before heating, was visually determined for both preparations. 

##### Chemical stability of CyA-loaded ASMP-Nano formulation 

To evaluate the chemical stability of CyA, three different batches of the selected Nano1HA_B_-CyA formulation were prepared following the method described in the paragraph 2.2.1. After preparation, the samples were filtered in sterile condition (0.2 μm, Phenex RC Syringe filter, Phenomenex^®^, BO, Italy) and packaged into different crimp vials. Then, they were incubated at 4 °C and 20 °C up to 90 days and the concentration of CyA in the samples was analysed over time by HPLC method after appropriate dilution with acetonitrile. The CyA detected in the samples was reported as percentage of initial amount and the coefficient of variation (CV%) was calculated according to the equation: (SD/mean) * 100, where SD represents standard deviation.

##### DSC and ATR-FTIR Analysis 

The presence of interactions among the different excipients and CyA utilised to prepare the selected nanomicellar formulation (Nano1HA_B_-CyA) was investigated by differential scanning calorimeter (DSC 4000, Perkin Elmer, Milan, Italy) and ATR-FTIR analysis (Agilent Cary 660 spectrometer with an ATR system containing a germanium crystal, Agilent Technology, Santa Clara, CA, USA). In both cases, the analyses were performed on a dried sample of Nano1HA_B_-CyA obtained by freeze-dried process of Nano1HA_B_-CyA formulation, on the pure samples of the drug (CyA), mucoadhesive polymer (HA) and single surfactants (VitE-TPGS and OPEE).

DSC analysis was carried out by using DSC, heating the samples under study from 30 °C to 200 °C at the constant rate of 10 °C per min. The samples of about 2–3 mg were directly weighed in aluminium pans, covered with aluminium lids and hermetically sealed using a pan press (Thermal Science, USA) The instrument was pre-calibrated with indium and the purging gas was nitrogen at a flow rate of 45 mL/min. Data were collected online using a Pyris software (13.3.1 version, USA). 

For ATR-FTIR analysis, each sample was placed in contact with the probe and spectra recorded from 900 to 4000 cm^−1^ at 4 cm^−1^ resolution with 32 scans, using an MCT detector. 

##### In Vitro CyA Release Studies 

In vitro release study of CyA from the selected Nano1HA_B_-CyA nanomicelles was investigated by dynamic dialysis method. Five hundred microliters of the Nano1HA_B_-CyA formulation was transferred into a closed dialysis bag (MWCO 3500 Da, Spectra/Pore 3 Dialysis Membranes, Spectrum labs, Breda, The Netherlands) and placed into borosilicate vials. The receptor medium containing ultrapure water (20 mL) was maintained at 32 °C, to simulate the temperature of the ocular surface, and stirred with a paddle running at 20 rpm, Sink conditions were maintained by replacing—at predetermined time intervals—1.0 mL of recipient phase with fresh solvent. All experiments lasted 30 h and were performed in triplicate.

As control, a 70% ethanolic solution (of CyA (EtOH-CyA, 1.0 mg/mL) was used. The amount of CyA in the receptor medium was determined by RP-HPLC. 

##### Cytotoxicity Assay 

A cytotoxicity test was performed on rabbit corneal epithelial cells (RCE) using the ready-to-use cell proliferation reagent WST-1. The RCE cells were plated at 5 × 10^3^ cells/well, in 96-well microtiter plates and treated with Nano1HA_B_-CyA, Nano1-CyA, and a reference CyA solution (DMSO-CyA), prepared by dissolving the drug in DMSO. All the formulations were suitably diluted in growth medium to the tested concentrations (1.0, 0.1, 0.01, 0.001, 0.0001% *w*/*v*). After 15 min of exposure the medium was removed, the cells were washed twice with DMEM/F12. Then, 100 μL of fresh growth medium and 10 μL of reagent WST-1 were added in each well, in accordance with the method reported by Chetoni et al [38]. The cells were incubated for 2 h at 37 °C; then, the microplate was thoroughly shaken for 1 min and the absorbance was measured at 450 nm using the microplate reader (Asys UVM 340, Biochrom, Cambridge, UK). The measurements were performed on at least four wells for each concentration and the cell viability was expressed as the percent optical density of treated vs. untreated control wells.

##### In vitro Permeation Studies of CyA through Ocular Reconstituted Tissue

COR-100 EpiCorneal^TM^ reconstituted tissues were chosen as biological substrate to evaluate the capability of CyA loaded into ASMP-Nano formulations to permeate through corneal tissues [39]. For the experiment, COR-100 EpiCorneal^TM^ reconstituted tissues were preliminary equilibrated overnight in culture medium (COR-100 ASY medium) at Standard Cell Culture Conditions (SCC, 37 °C, 5% CO_2_) in line with the Mattek protocol. Then, the tissues were transferred into two different 12-well plates and equilibrated with 500 µL of Krebs Ringer Bicarbonate buffer (KRB, pH 7.4) for 30 min before the beginning of each treatment. For the test, 100 µL of selected formulation Nano1HA_B_-CyA, and 100 µL of each reference formulation were applied on tissue surface (apical layer). The reference formulations were Nano1-CyA formulations (see Table 1), and the formulation available on the European market contained 0.1% *w*/*w* of CyA (Ikervis®, Santen Oy, Tampere, Finland). The plates were incubated at SCC on a horizontal plate shaker (Incubating Microplate Shaker, VWR^®^, Philadelphia, USA) able to determine a slight agitation and reduce the possibility that the permeated molecules of drug in proximity of the apical layer will hinder drug permeation through the barrier. At predetermined time intervals (0.5, 1, 2, 3, 4 h), 300 µL of KRB-receiving medium were collected to analyse CyA content and replaced with the same volume of fresh medium. The experiment was performed in triplicate.

Moreover, the EpiCorneal^TM^ transepithelial electrical resistance (TEER) was measured to evaluate the integrity of the corneal barrier at the beginning and at the end of each permeation experiments. In detail, the initial resistance (t = 0 h; TEER-1) was measured after the preliminary treatment, immediately after filling the wells with 300 µL (donor phase) and 500 µL of KRB buffer solution (receiving phase) using an epithelial volt-ohm meter (EVOM) and the EndOhm-12 chamber (World Precision, Sarasota, FL, USA). Furthermore, the resistance of the tissues was measured for each sample at the end of the permeation study (t = 4 h; TEER-2) in the same conditions. The resistance was measured for three different samples of EpiCorneal^TM^ tissues. Statistical differences related to barrier integrity (TEER) between TEER-1 and TEER-2 were evaluated using Student’s two-tailored unpaired *t-*test (Prism 8 software). The evaluation included calculation of the mean ± standard error (S.E.). Differences were considered significant at *p* < 0.05. 

##### DSC analysis and ATR-FITR of HA-FITC Derivative

DSC and ATR-FITR analysis was carried out on the samples of the synthetized HA-FITC derivative and of the single ingredients, HA, FITC according to the experimental conditions already described above. 

#### 2.2.5. Ex Vivo Study on Isolated Cornea of Assembling Surfactants-Labeled Mucoadhesive Polymers Nanomicelles (ASLMP-Nano) Formulation

The animals were sacrificed intravenous administration of an overdose of sodium pentobarbital solution (Pentothal sodium®, Gellini Farmaceutici, Milan, Italy) in the ear marginal vein, the eyes were enucleated and the corneas, with a 2 mm perimeter of sclera, were immediately excised and mounted in a plexiglass perfusion cell with a corneal area available for diffusion of 0.78 cm^2^ [40]. Preheated (35 °C) pH = 6.8 glutathione bicarbonate Ringer buffer (GBR) was added to both the epithelial (1.0 mL) and the endothelial (5.0 mL) compartments. To ensure oxygenation and agitation, an O_2_/CO_2_ (95:5) mixture was bubbled through each compartment at a rate of 3/4 bubbles per second. After 10 min of equilibration, the solution on the epithelial side was withdrawn and substituted with 1.0 mL of 20% *w*/*w* labelled Nano1HA_B_FITC-CyA formulation in GBR. At predetermined time intervals, 0.5 mL samples were withdrawn from the receptor compartment, replaced with an equal volume of GBR to promote the penetration of fluorescent probe into the depth of corneal tissue.

At the end of the permeation experiment, the treated corneas were fixed with 4% paraformaldehyde in 0.1 M phosphate buffer saline (PBS, pH 7.4). After several washings in PBS, they were cryoprotected in a solution of 20% sucrose in PBS, frozen and stored at −80 °C. Cross sections (15 µm thick) were obtained with a cryomicrotome (Kryostat 1720, Leitz Wetzlar), mounted on gelatin-coated slides with Vectarshild mounting medium with DAPI us nuclear counterstain (Vector Lab., Inc. Burlingame) and analysed by laser microscope (Model Nikon Ni-e Microscope equipped with NIS-Elements Br Software (Nikon Instruments, Florence, Italy). 

#### 2.2.6. In Vivo Evaluation of Precorneal Residence Time of CyA

Fifty microliters of CyA loaded nanomicellar formulations (Nano1HA_B_-CyA and Nano1-CyA) were instilled into conjunctival sac of the rabbit eye and tear fluid samples were taken from the lower marginal tear strip, using a 1.0 μL disposable glass capillary (Microcaps, Drummond Scientific, NJ, USA) at 1, 3, 5, 10, 20, 30 min after instillation [40]. The collected sample was then put into micro-tubes Eppendorf®, and the capillary rinsed with ultrapure water (1.0 μL). The resulting sample was further diluted with 98 μL of acetonitrile, obtaining a final sample volume of 100 μL. Then, after centrifugation at 13,000 rpm for 5 min (Micro CL 17, Thermo Fisher, Italy), the supernatant samples (50 μL) were analysed by HPLC with appropriate calibration curve. A commercial 0.1% *w*/*v* CyA emulsion (Ikervis®) was used as control.

The apparent first order elimination rate constants (K_e_) of CyA from the tear fluid and the corresponding half-lives (t_½_= 0.963/K_e_) were calculated from linear phase of [log tear fluid concentration] *vs.* time plots [41]. The Areas Under Curve values (AUC) were calculated from 1 min to 30 min after instillation from appropriate graph applying the linear trapezoidal method. 

For all the formulations, the tear fluid concentration of CyA at the different time points were compared for statistical significance (*p < 0.05*) using Student’s two-tailored unpaired *t-*test (Prism 8 software). The evaluation included calculation of the mean ± standard error (S.E.).

#### 2.2.7. HPLC Analytical Method 

The quantitative determination of CyA was carried out by a partially modified reverse phase HPLC (RP-HPLC) method [42]. The apparatus consisted of a Shimadzu LC-20AD system with a UV SPD-10A detector equipped with an autosampler CBM-20A and a computer integrating system (Shimadzu Italia s.r.l., Milan, Italy). The injection valve was a Rheodyne with a capacity of 20 μL and the column were a Gemini-C_18_ (250 mm × 4.6 mm, Phenomenex, Torrance, CA, USA) having a porosity of 110 Å and a particle size of 5 μm. The mobile phase (acetonitrile: water: methyl tert-butyl ether, 52:43:5 mixture containing the 0.1% *v*/*v* of 85% phosphoric acid), was heated at 80 °C and delivered at a flow rate of 1.0 mL/min. The detection wavelength was 210 nm and the retention time of CyA in this chromatographic condition was 32 min. 

The amount of drug in the samples was determined by comparison with appropriate external standard curves. The calibration curves were obtained applying the least square linear regression analysis by using Prism 8 software (GraphPad Software 164 Inc., San Diego, CA, USA).

For in vitro studies, a calibration curve was obtained by dissolving the drug in acetonitrile (stock solution) and then diluting with the related medium (ACN, water or KBR). The related parameters of each calibration curve are summarised in Table 2.

For in vivo study, a stock solution of CyA in acetonitrile was progressively diluted with the same solvent to produce a series of standard solutions (concentration range: 0.05–6.50 μg/mL). Ninety-eight microliters of each standard solution were added to rabbit rinsed tear fluid (2 μL) and the final solution was then centrifuged at 13,000 rpm for 5 min and 20 μL of supernatant analysed. 

The limit of determination (LOD) and limit of quantification (LOQ) were estimated on the basis of the standard deviation of the response and the slope, accordingly, with ICH Q2 (R1) (ICH, Harmonised Tripartite Guideline – Validation of analytical procedures: text and methodology – Q2 (R1), (2005) Geneva, Switzerland.).

## 3. Results 

### 3.1. Optimisation of Assembling Surfactants-Mucoadhesive Polymers Nanomicelles (ASMP-Nano)

**Figure 1:**
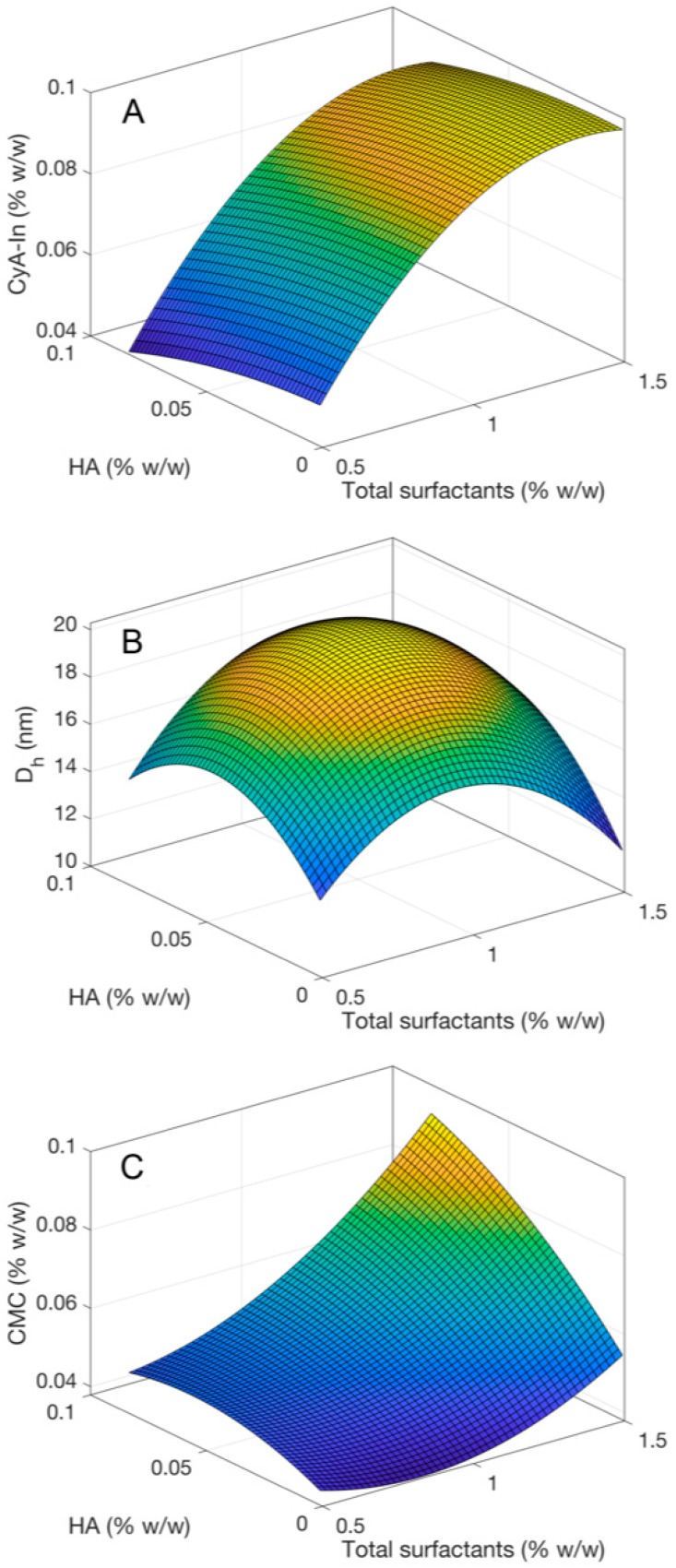
Results of DOE surface analysis in term of percentage of CyA solubilised (CyA-In) (**A**), Hydrodynamic Diameter (D_h_) of the nanomicelles (**B**) and Critical Micellar Concentration (CMC) (**C**).

Continuous response surfaces of two independent variables (X1 = total percentage of mixture of surfactants Vit E-TPGS and OPEE 2.25:1 constant ratio, and X2 = percentage of HA added to the formulations) were calculated in order to select the most efficient nanomicellar formulation. The response surfaces were quadratic in both the considered independent variables and were based on the least square method on the points provided in the Design of Experiment (DOE). Three dependent variables were chosen as goal functions: CyA solubilisation (CyA-In), hydrodynamic diameter (D_h_) and CMC. The related results are reported in Figure 1A–C, respectively, and are summarised in Table 3. The CyA solubilisation degree, shown in Figure 1A, was found to depend mainly on the amount of surfactants, while the influence of HA can be considered negligible. In particular, a high concentration of surfactants, more than 0.8% *w*/*w*, was necessary to obtain a CyA-In increase. Indeed, the formulations with the higher percentages of total surfactants, i.e., 1.5 and 1.0% *w*/*w*, lead to a concentration of solubilised CyA in the range 0.082-0.105% *w*/*w*. On the contrary, a gradual reduction in the concentration of solubilised CyA was observed for the lower surfactants’ percentages. CyA-In values for the latter case ranged from 0.041 and 0.054% *w*/*w*.

The formulations showed a mean value of the hydrodynamic diameter Dh between 12.25 nm and 22.95 nm (Figure 1B). From an in-depth analysis of the numerical results summarised in Table 3, the range of the mean value of Dh can be further narrowed to 12.25–16.70 nm considering eight of the nine formulations. A significantly different mean value of Dh has been found when the total surfactants concentration and HA concentration were 1.0% and 0.1% *w*/*w*, respectively. Moreover, seven of the nine formulations presented a low value of the standard deviation (below 3.2 nm). The highest standard deviation values are found for the Nano1HA_C_-CyA (surfactants/HA: 1.0/ 0.1%) and Nano0.5HA_B_-CyA (surfactants/HA: 0.5/0.01%) formulations with the Dh highest values.

Moreover, the response surface for CMC, shown in Figure 1C, is quite constant for low and moderate values of HA concentrations, whereas a rapid increase of CMC values was observed for the highest HA and surfactant concentrations. It is reasonable to infer that the aggregation and formation of nanomicelles in presence of HA occurs with the highest surfactants’ concentration.

The data above obtained demonstrated that the more promising nanomicellar formulation in term drug entrapment capacity, mean size distribution and CMC, was Nano1HA_B_–CyA, containing a combination of a 1.0% *w*/*w* total surfactant and 0.01% *w*/*w* HA. The selected Nano1HA_B_–CyA formulation showed a percentage of CyA-In, mean diameter and CMC equal to 0.105% *w*/*w*, 14.41 ± 0.41 nm and 0.045% *w*/*w*, respectively. Although a similar ability to solubilise CyA was observed for the binary mixtures of surfactants at higher concentration 1.5% *w*/*w*, the preparation containing the lower concentration of surfactant mixture (1.0% *w*/*w*) sufficient to solubilise the required CyA (0.1% *w*/*w*) was selected to minimise the risk of ocular side effects, such as irritation or inflammation.

Although a similar ability to solubilise CyA was observed, the preparation containing the lower concentration of surfactant mixture was selected, to minimise the risk of ocular side effects.

### 3.2. Physico-Chemical Characterisation of the Selected Nanomicellar Formulation (Nano1HA_B_–CyA)

#### 3.2.1. Determination of the Cloud Point

**Figure 2:**
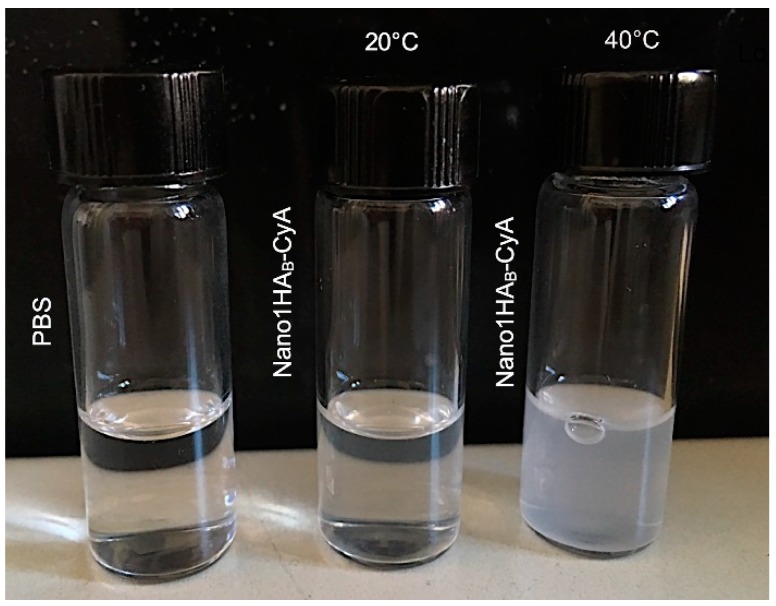
Visual appearance of the nanomicelles: phosphate buffer solution (PBS), Nano 1HA_B_-CyA (total surfactant amount 1.0%; HA 0.01%) at 20 °C and 40 °C.

The cloud point (CP) was one of the physico-chemical parameters measured for the selected Nano1HA_B_–CyA formulation and for the blank reference Nano1HA_B_. A high absorbance values corresponding to 0.158 ± 0.021 and 0.035 ± 0.006 were recorded at 400 and 500 nm, respectively, when the Nano1HA_B_–CyA formulation was heated at 40 °C. On the contrary, the reference unloaded CyA nanomicellar formulation (Nano1HA_B_) showed a significant absorbance value exclusively when the temperature was increased up to 80 °C (0.216± 0.001 and 0.071 ± 0.002 at 400 and 500 nm, respectively). The CP values for unloaded drug formulations were generally 30 to 40 °C higher than those measured for the same formulations containing the drugs [28]. The uptake of CyA into core of the nanomicelles decreases the hydrophobic interaction among the hydrocarbon tail groups of TPGS molecules, reducing the CP temperature [43,44]. The heating over the CP temperature led to disruption of the nanomicelles structure with release of the drug to the surrounding aqueous environment. This destabilisation produced the formation of a cloudy/milky dispersion. A picture of the formulated nanomicelles at different experimental conditions compared with a phosphate buffer solution (PBS) are presented in Figure 2. The difference in the CP temperature of CyA-loaded and unloaded nanomicelles can be considered as an indication of successful incorporation of the drug inside the nanomicelles. In addition, the CP of Nano 1HA_B_–CyA formulation was sufficiently high to potentially provide a good stability after instillation onto the ocular surface (temperature is 30–32 °C). Furthermore, nanomicelles were regenerated in about 1 min and 10 min for Nano1HA_B_ and Nano1HA_B_-CyA, respectively, when the formulation was brought down to room temperature (20 °C), resulting in optically clear dispersions. The relatively long time required to regenerate the nanomicelles could be ascribed to the long relaxation time due to the presence of hyaluronic acid.

#### 3.2.2. Osmolality, pH, Yield, Entrapment and Loading Efficiency 

All the formulations showed osmolality and pH values in the physiological range, between 280-318 mOsm/kg and 6.6–7.3, respectively. The selected formulation and the reference showed similar osmolality and pH values, as evidenced in Table 4. Indeed, the osmolality were 316 ± 1.03 mOsm/kg and 316 ± 0.33 mOsm/kg, while pH was 6.8 ± 0.05 and 6.8 ± 0.04 for Nano1HA_B_–CyA and Nano1HA_B_ formulations, respectively. 

The nanomicellar selected formulation showed a good yield, approximately of 95%, confirming the successful of the preparation method.

The entrapment and loading efficiency were also reported in Table 4. The percentage of CyA entrapped into Nano 1HA_B_–CyA was of 77.66 ± 1.77% *w*/*w*, which is encouraging since an amount of drug solubilised in the selected formulation (0.105% *w*/*w*) corresponding to the commercial product, Ikervis^®^, was obtained.

#### 3.2.3. Stability studies of CyA in Nano 1HA_B_-CyA Formulation

The ability of Nano1HA_B_-CyA to maintain the chemical stability of its cargo was monitored over time up to 90 days, by analysing the amount of CyA remaining when the formulation was stored at 4 °C and 20 °C. In both cases, the formulation remained clear. The percentage of CyA (mean ± SE) remained in the range of 95.17 ± 0.41–97.19 ± 0.35 (CV%= 0.21–1.54) and 95.55 ± 0.05–97.45 ± 1.25% (CV% = 0.07–1.81) *w*/*w* at 4 °C and 20 °C, respectively. These results demonstrate no significant change in stability over the time since CV% remained under 5% with no alteration in the formulations physical appearance, meeting the acceptance criteria of international guidelines [Eudralex 3AQ11a; Guideline: Specifications and Control Tests on the Finished Product - 75/318/EEC as amended; EMA (2015) Guideline on Bioanalytical Method Validation, European Medicine Agency, London, UK].

#### 3.2.4. DSC and ATR-FTIR Analysis of Nano 1HA_B_-CyA

**Figure 3:**
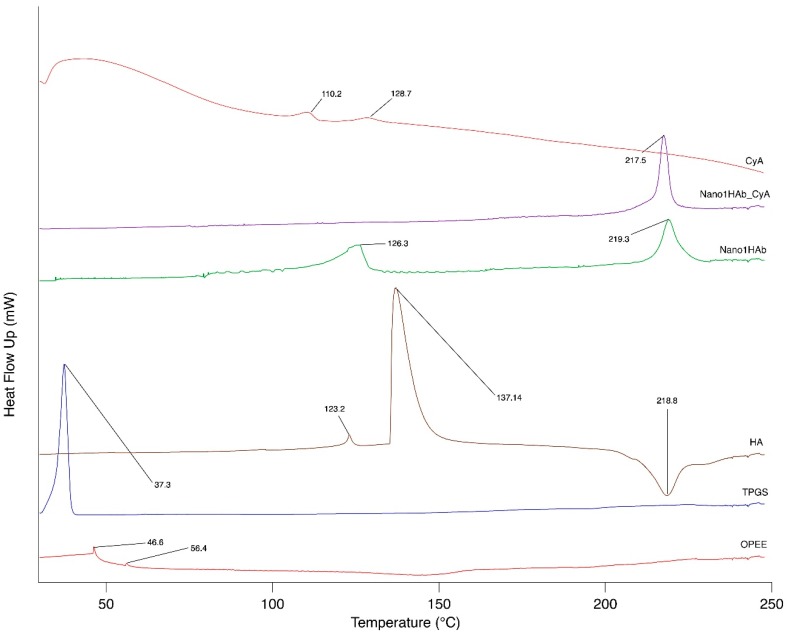
DSC thermograms of VitE-TPGS, OPEE, HA, CyA and freeze-dried nanomicelles with and without CyA, Nano 1HA_B_-CyA and Nano1HA_B_, respectively (total surfactant amount 1.0%; HA 0.01%).

**Figure 4:**
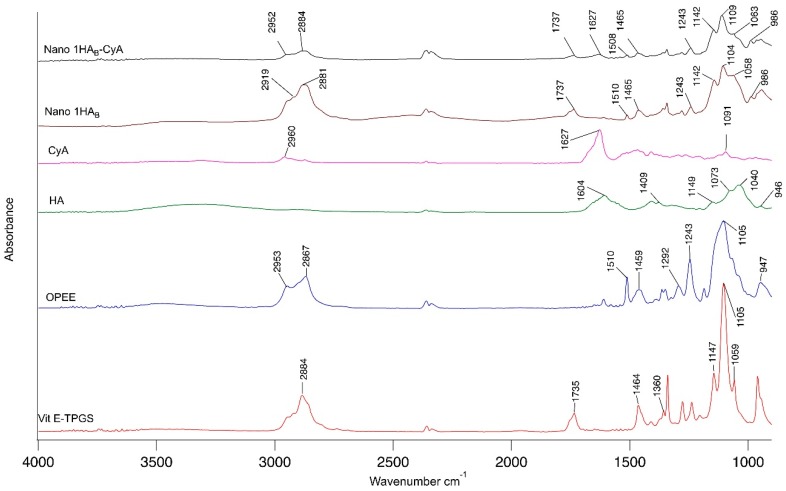
ATR-FTIR spectra of VitE-TPGS, OPEE, HA, CyA and freeze-dried nanomicelles with and without CyA, Nano 1HA_B_-CyA and Nano1HA_B_, respectively (total surfactant amount 1.0%; HA 0.01%).

DSC thermograms and FTIR spectra of the starting materials (Vit E- TPGS, OPEE, HA and CyA), and of the freeze-dried nanomicellar formulations with and without CyA (Nano1HA_B_-CyA and Nano1HA_B_) are illustrated in Figure 3 and Figure 4. The freeze-drying process was carried out in a Virtis apparatus (VirTis Wizard 2.0, SP Scientific) in the following experimental conditions: a freezing phase with pressure 400 torr, temperature—38 °C, rate 0.6 °C/h, extra freeze time 120 min; b-primary drying phase with a pressure 100 torr, temperature—38 to 0 °C, rate 2.1 °C/h; c- secondary drying phase at a pressure of 50 torr, temperature—0 to 25 °C, rate 5.0 °C/h, extra drying 27 °C for 60 min [45].

The obtained freeze-dried samples were stored in a desiccator up to the analysis.

According to literature data [46], DSC thermogram of pure CyA highlighted that it is both in crystalline (110.2°C) and amorphous (128.7 °C) forms. In both nanomicellar formulations (Nano1HA_B_ and Nano1HA_B_-CyA), thermal transition of the surfactants disappeared and there was an endothermic peak at 219.3 °C for Nano1HA_B_ shifted to 217.5 °C for Nano1HA_B_-CyA

DSC analysis of Nano1HAB-CyA did not highlight any peaks of transition corresponding to the drug. This behaviour could, on one hand, confirm the effective CyA encapsulation into the nanomicelles; on the other this could be due to a small amount of encapsulated drug that did not allow appreciating its transition. However, further confirmation of the drug encapsulation in the entanglement of the polymer in Nano1HA_B_-CyA can be given by the disappearance of the specific transitions of the HA. HA could act as solvent for the quantity of CyA entrapped into the nanomicelles, leading to its dissolution.

FTIR spectrum of Vit E-TPGS displays the characteristic bands at ~ 2884 cm^−1^ (C-H stretching) and the carbonyl band at ~ 1735 cm^−1^. At 1050–1250 cm^−1^ and at ~1464 cm^−1^, both the surfactants (Vit E- TPGS and OPEE) show the typical C–O stretching and CH_2_ scissoring bands, respectively. The FTIR spectrum of HA shows the characteristic carbohydrates bands at ~1149, 1073, 1040 and 946 cm^−1^; the polymer bands at ~ 1600 and 1400 cm^−1^ were assigned to the asymmetric and symmetric stretching modes of planar carboxyl group, respectively [47]. The anhydrous polypeptide CyA shows a characteristic band at ~ 1627 cm^−1^ (C–C stretching) for the amide bonds. 

FTIR spectrum of Nano1HA_B_ formulation showed the typical bands of both surfactants forming the nanomicelle. The absorption band of the selected Nano1HAb-CyA did not show interference with the characteristic peaks of the drug, indicating the compatibility among the components and the presence of CyA in the nanomicelles. Typical bands of HA are overlapping with the surfactants bands; this may be due to the relatively low polymer percentage present in the formulation (0.01%w/w) compared with the total surfactants content (1.0%). 

#### 3.2.5. DSC and FTIR Analysis of HA-FITC

**Figure 5:**
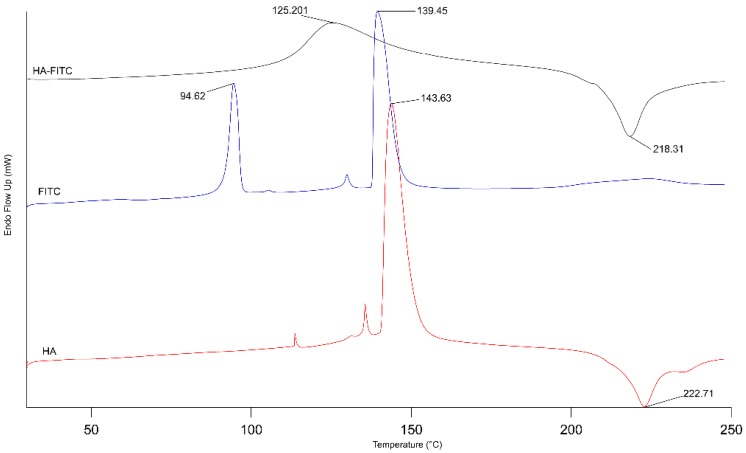
DSC thermograms of HA, FITC and HA-FITC.

**Figure 6:**
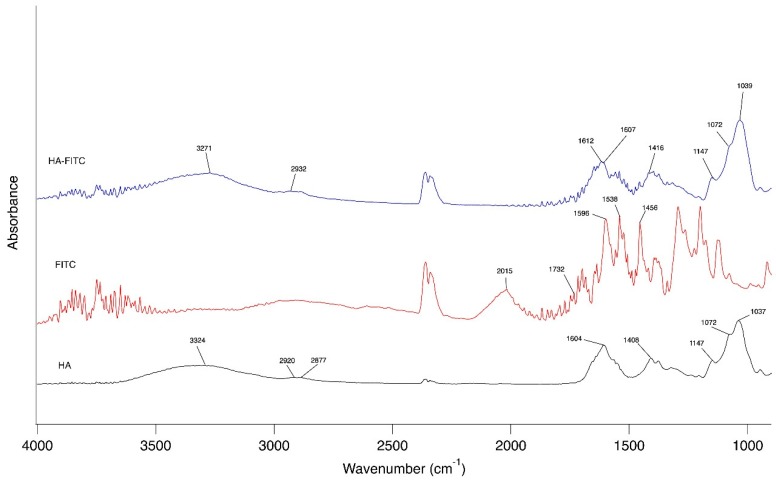
FTIR spectra of HA, FITC and HA-FITC.

The fluorescent derivative of HA (HA-FITC), used to prepare the fluorescent nanomicelles, was analysed by DSC and FTIR analysis. The results are reported in Figure 5 and Figure 6 Regarding to DSC analysis, a melting transition at 143.63 °C, followed by an exothermal peak at 222.71 °C was observed for HA. Moreover, a double endothermic transition at 94.62 and 139.45 °C was detected for FITC. The fluorescent derivative HA-FITC has shown a single shift at a lower temperature of HA transition peaks of about 18 °C for the endothermic transition and of about 4 °C for the exothermic peak, without any other evident transitions related to FITC, suggesting that the two components are no longer a pure physical mixture. This hypothesis was confirmed by FTIR spectrum (Figure 6). FITC shows a strong isothiocyanate characteristic peak at 2015 cm^−1^ as well as a carbonyl group of lactone at around 1732 cm^−1^ and absorption peaks in the range between 1596 and 1456 cm^−1^ related to benzene ring stretching vibration. Compared to FITC, the isothiocyanate characteristic peak at 2015 cm^−1^ disappeared in the spectrum of HA-FITC, suggesting the involvement of this group in the addition reaction with the hydroxyl group and then the success of the reaction.

### 3.3. Cytotoxicity Assay 

**Figure 7:**
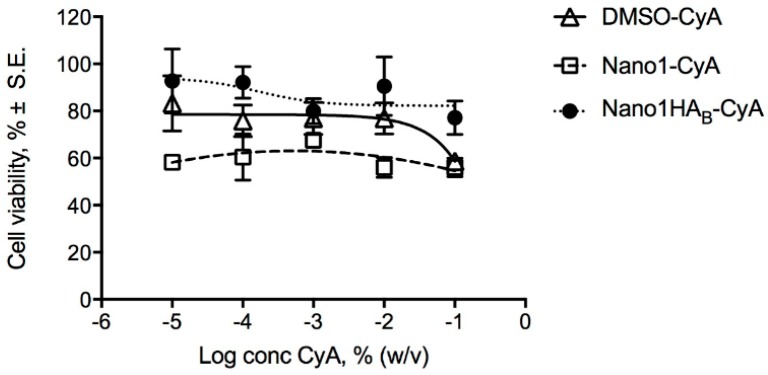
Cytotoxicity of DMSO-CyA solution, Nano 1-CyA and Nano 1HAB-CyA formulations on rabbit corneal epithelial cells.

The treatment of the cell line with the different reference preparations did not reveal a dose-dependent toxic effect. Cell viability was over 50% for all tested concentrations, as shown in Figure 7. The CyA solution (DMSO-CyA) appeared to maintain cell viability of around 75%, except the highest concentration, which produced 58% in viability. The nanomicellar system, where surfactants and drug were combined (Nano1-CyA), produced a toxic effect, reducing cell viability to 56%–58%, independent of drug concentration. The addition of HA (Nano1HAB-CyA) appeared to offset the negative effect of the nanomicellar composition resulting in a cellular viability of 77% to 93% for the highest and lowest CyA concentrations, respectively. These results confirm the protective effect of HA, which has been shown to reduce the toxicity of both CyA and surfactants as reported by Debbash et al. [48]. The protective effect of hyaluronic acid against damage of the corneal epithelium and its ability to stimulate epithelial migration has been already reported [49,50,51,52,53]. However, it is surprising that the small amounts of HA in the current nanomicelle formulations promoted such a reduction in cytotoxicity. 

### 3.4. In Vitro Drug Release Studies

**Figure 8:**
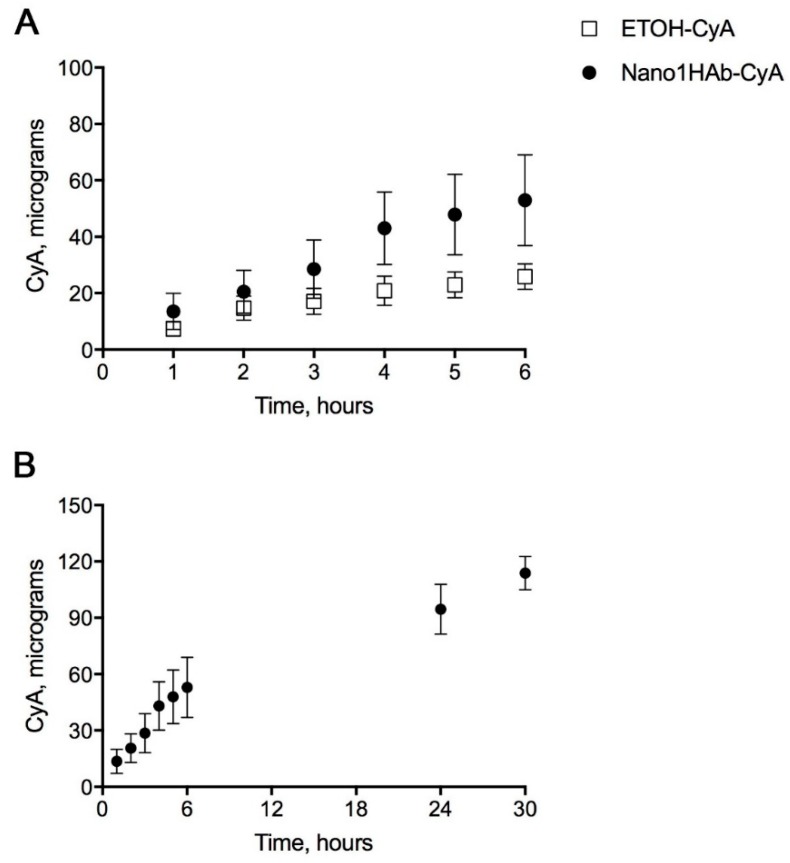
In vitro drug release profile of CyA from Nano1HA_B_-CyA formulation and reference solution (ETOH-CyA). (**A**) 6 h experiment and (**B**) 30 h experiment.

The Nano1HA_B_-CyA formulation showed a rapid CyA diffusion, releasing in 6 h twice the amount of CyA (52.96 ± 16.05 μg) compared to the EtOH-CyA reference solution (25.89 ± 4.51 μg), suggesting a positive influence of the nanomicellar structure on the release of the drug, which does not seem to depend on whether the drug is in a molecular or colloidal dispersion, as depicted in Figure 8. Moreover, the release of CyA from Nano1HA_B_-CyA was followed up to 30 h, showing that the formulation under study was able to release up to 113.87 ± 8.98 μg of the drug. Based on the data, CyA release from individual micelles is approximately 16% and 19% in 24 and 30 h, respectively. Goyal et al. [54] found that CyA-loaded TyroSpheres released about the 23% of drug in one day. It could be assumed that the low CyA release from the nanostructured vehicle was related to drug-polymer high compatibility. The compatibility between drug and polymer is an important issue in the effectiveness of polymeric delivery systems including micellar structures [55]. In general, the better the affinity between a drug and polymer, the higher the loading content and the slower the drug release. Our experimental data supported these concepts and are further confirmed by DSC and ATR-FTIR results (Figure 3 and Figure 4). 

### 3.5. In vitro Permeation of CyA through Ocular Reconstituted Tissue 

EpiCorneal^TM^ tissue was a suitable model to study CyA permeation through corneal epithelium. More precisely, it represents the apical surface of the epithelium. This tissue contributes to the overall barrier properties of the cornea that typically represents the limiting structure relating to the permeability of ophthalmic drugs. EpiCorneal^TM^ is a three-dimensional (3D) corneal tissue model derived from human corneal epithelium containing tight-junctions and desmosomes, expressing tight junction proteins in the apical cell layers and developing TEER above 1000 Ohm/cm^2^. The in vitro permeation studies of CyA using EpiCorneal^TM^ tissue were performed on the two nanomicellar formulations (Nano1-CyA and Nano1HA_B_-CyA) and on the commercial emulsion Ikervis^®^, chosen as reference. The CyA permeation through the Epicorneal tissues can be excluded in consideration of the good sensibility of the chosen analytical HPLC method. Indeed, the calculated LOQ was 50 νg/mL, assuring the lack of trace of drug in the receiving phase of the permeation test.

Before starting the permeation experiments, TERR of EpiCorneal^TM^ was measured to confirm the integrity of reconstituted tissue. At the end of the drug permeability experiments (after 4-hour of incubation), changes in 3D corneal tissue barrier integrity were again evaluated by measuring TEER. The reduction of the resistance was of 77.09% ± 6.09 (S.E., *n* = 6) and 92.76% ± 6.95 (S.E., *n* = 6) for Nano1CyA and Nano1HA_B_CyA formulations, respectively. Statistically significant difference between TEER reduction was detected for EpiCorneal^TM^ tissues treated with Nano1HA_B_CyA and Ikervis (65.00% ± 6.44, S.E., *n* = 6) (*p* = 0.04, *t*-test). These findings demonstrate the high biocompatibility of the nanomicellar formulations with the ocular tissues. The presence of irritating substance in the eye drop decreased more significantly for the TEER values at the end of permeation study through Epicorneal^TM^ tissue. Our unpublished data evidenced that there was a TEER reduction after 4 h of permeation test, performed with a commercial multidose eye drops containing more than 100 ppm of benzalkonium chloride was of about 50%.

Nano1HA_B_-CyA resulted in less reduction in TEER value after 4 h of permeation experiment compared to Nano1-CyA, highlighting the protective effect of hyaluronic acid. These results were in agreement with the cell viability results, where the protective effect of hyaluronic acid had been already demonstrated.

### 3.6. Ex Vivo Corneal Permeation Studies using the Fluorescent Nano1HA_B_FITC-CyA Nanomicelles 

The study of permeation was performed with Nano1HA_B_FITC-CyA after determining the hydrodynamic diameter and the CyA concentration into the formulation. The Nano1HA_B_FITC-CyA nanomicelles were 11.5 nm ± 0.23 (SD) in size (D_h_) and contained 0.11% ± 0.003 *w*/*w* of CyA. The amount of CyA was in the same order of magnitude than those detected for Nano1HA_B_-CyA.

**Figure 9:**
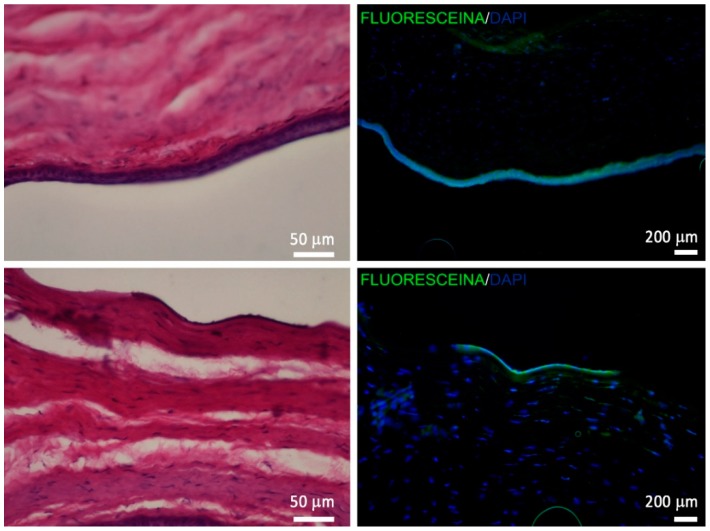
Microscopy images of corneal tissue of albino rabbit eyes after treatment with Nano1HA_B_FITC-CyA.

At the end of the permeation experiments, the microscopic imaging analysis performed on the fixed tissue and reported in Figure 9 showed no alteration signs of corneal structure and an evident fluorescence on epithelium limiting the cornea. In particular, a visible fluorescence was found on the external stratified squamous basement epithelium (upper images) and on the internal flat endothelial cells.

This result suggests the possibility of interaction between nanomicelles and apical cells of the ocular tissues and this behaviour is probably promoted by the mucoadhesive properties of HA. The presence of HA in the structure of the nanomicelles could facilitate the CyA delivery towards inner layers of the eye barriers. 

### 3.7. In Vivo Studies: Tear Fluid Pharmacokinetic in Rabbits

**Figure 10:**
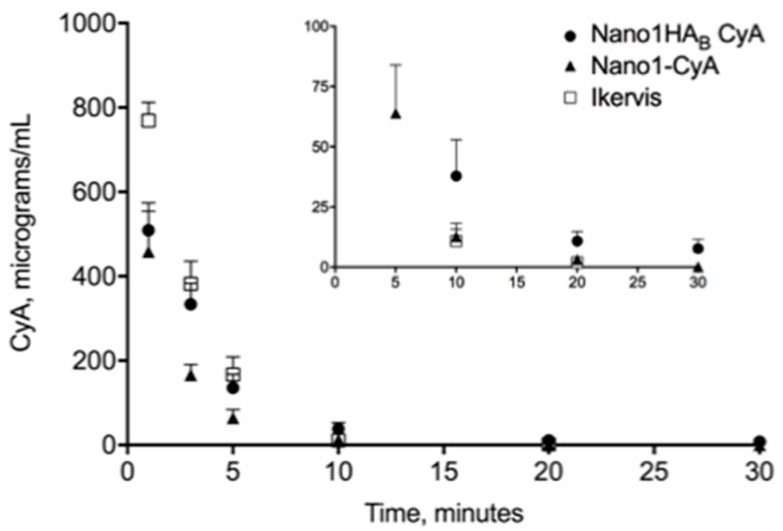
Ocular pharmacokinetic profiles on rabbits after instillation of experimental (Nano1-CyA and Nano1HA_B_-CyA) and commercial (Ikervis) products.

The tear fluid CyA concentration (mean ± S.E., *n* = 6) *vs.* time profiles resulting from administration to rabbits of the nanomicellar formulations (Nano1HA_B_-CyA and Nano1-CyA) and of the commercial emulsion named Ikervis^®^ containing 0.1% of CyA, chosen as reference, are presented in Figure 10. The main pharmacokinetic parameters of CyA in tear fluid are summarised in Table 5, where the concentration of CyA at 1 min after instillation (C_1min_), area under the curve (AUC), elimination rate constant (K_e_) and half-life time (t_1/2_) are reported. 

In all cases, the drug concentration in tear fluid was typically high after instillation (C_1min_ = 769.16, 508.99 and 458.18 μg/mL for Ikervis^®^, Nano1HA_B_-CyA and Nano1-CyA, respectively) followed by a rapid decrease. Nanomicelles exhibited better residence potentials on the ocular surface compared to commercial eye drops, Ikervis^®^; nevertheless, the CyA levels in tear fluid at early stages for Ikervis were higher than the nanomicellar formulations. Indeed, the elimination rate constants of CyA from tear fluid of rabbits (K_e_) was 1.7 times and more than four times smaller for Nano1-CyA and Nano1HA_B_-CyA formulations, respectively, compared to Ikervis^®^. Statistically significant differences were observed in C_1min_ values between Ikervis and both nanomicellar formulations, but exclusively Nano1HA_B_-CyA produced K_e_ and t_1/2_ values were significantly different (*p* < 0.05) from Ikervis^®^, ascribable to the high affinity of the nanomicellar formulation containing hyaluronic acid for the ocular surface. Furthermore, the selected Nano1HA_B_-CyA formulation determined an improvement of CyA bioavailability (AUC) in tear fluid of about 1.5-fold compared to Nano1-CyA (*p* < 0.05). This behaviour confirmed the hypothesis for which the presence of hyaluronic acid in Nano1HA_B_-CyA formulation can contribute to extended residence time in the precorneal area by virtue of its muco-adhesive properties as supported by the data of elimination rate constant (K_e_) and t_1/2_. 

Similar profiles in the elimination of CyA from the tear fluid of rabbits after instillation of a nanomicellar formulation and Restasis^®^ emulsion, used as reference, was observed by Di Tommaso et al. [22]. Even if the concentration of the formulations and the instilled dose were lower compared to those utilised in our study, the ratio between the two area under curves (AUC nanomicelle/AUC reference) was of the same order of magnitude as those found in ours: 1.01 *vs.* 1.18. By analysing the pharmacokinetic data at 30 min of Di Tommaso et al. [22], the experimental formulation produced an increase of CyA concentration in tear fluid by 1.67-fold compared to Restasis^®^. The Nano1HA_B_-CyA formulation, instead, maintained, at the same time point, a three-fold higher CyA concentration (7.7 μg mL^−1^) compared with Ikervis^®^**,** demonstrating higher affinity of the nanomicellar formulation for the ocular tissues.

## 4. Discussion

The main factors behind the poor ocular bioavailability of drugs are mainly due to precorneal elimination factors, such as tear production and/or drainage through nasolacrimal duct that determine the loss of the majority of simple solution eye drops instilled into the conjunctival sac and by the presence of ocular barriers able to prevent drug permeation/absorption into the ocular tissues. An acceptable balance between maintaining a therapeutic concentration of drug in the precorneal area for a long time and minimising absorption through the eye barriers is the goal pursued with the design of Assembling Surfactants-Mucoadhesive Polymers Nanomicelles (ASMP-Nano) for the treatment of ocular surface pathologies. The main stream commercially available formulations range from viscous or gel-forming vehicles, nano-structured vehicles, up to solid drug delivery systems. Nanomicelles have been proposed as potential ocular carriers both for their capability to entrap lipophilic drugs and to protect them from precorneal elimination or to promote drug uptake into intraocular compartments. The interest in nanomicelles has recently increased with the recent FDA approval of Cequa^®^, a cyclosporine based liquid nanomicellar formulation indicated for the treatment of dry eye syndrome. The topical instillation of cyclosporine nanomicelles resulted in an expansive distribution into cornea and conjunctiva the target tissues for the treatment of dry eye syndrome [56]. In addition, the biopharmaceutical potential of nanomicelles has been enhanced by preparing active targeting nanomicelles capable of promoting active transport of drugs in the posterior segments of the eye [57].

Our results demonstrated the possibility to develop a mixed nanomicellar formulation based on non-ionic surfactant and mucoadhesive polymer capable of delivering therapeutic amounts of CyA amount required for treatment of dry eye syndrome. The amount of encapsulated CyA in the AMSP-Nano formulation was double that of Restasis® (0.05%) and the same as Ikervis® (0.1%) and Cequa® (0.09%). 

Pursuing a simple method of preparation, nanomicelles with preferably small diameters (about 14 nm), good stability at the physiological temperature (as demonstrated by CP values) were prepared. Such nano-sized range micelles can interact with cellular barriers favouring the uptake and the accumulation of the loaded CyA into corneal cells. Indeed, the distribution of fluorescence into the epithelium limiting the cornea up to the external stratified squamous basement epithelium and onto the internal flat endothelial cells after the ex vivo permeation studies support this hypothesis. This behaviour could depend on the interpenetration of the nanomicelles into the mucous surrounding of the epithelial corneal cell, which could explain the low concentrations of CyA in the tear fluid few minutes after instillation. In the case of the reference emulsion (Ikervis^®^), all the instilled dose of CyA was immediately available in the tear fluid while the AMSP-Nano formulation remained entrapped into the mucous causing a slow release of loaded CyA as evidenced by in vitro release test. Furthermore, no significant cytotoxicity on the rabbits’ corneal epithelial cell line was observed with AMSP-Nano formulation; the protective effect could be due to the presence of hyaluronic acid (formulation Nano 1HAB-CyA) that maintain a cell viability of more than 80% for all the tested concentration. A similar effect was reported by Xiaoyue et al. [57] for a nanomicellar carrier of dexamethasone. 

The potential of the ASPM-Nano for ocular drug delivery of CyA were demonstrated by the in vitro permeability and in vivo pharmacokinetics results. Specifically, the nanomicellar carrier did not enhance the permeation of CyA to any extent through reconstituted corneal epithelium (lower than LOQ values of 50 ng/mL), yet it showed a tendency to prolong the CyA release in the precorneal area. This behaviour could be mainly ascribed to the mucoadhesive polymer HA as well as to the sustained release of the drug from the non-structured system, pointed out by the study of release. The nanomicelles did not cause any evident ocular irritation when instilled into rabbit eye, suggesting a good tolerability. The developed formulations did not interfere with the tear production, nor did they induce any discomfort which is experienced with the commercial product due to its a lipophilic vehicle, which is poorly tolerated by the ocular surface, The bioavailability of CyA loaded into our nanomicellar formulation was of the same order of magnitude of the reference Ikervis^®^, yet the elimination rate constant was smaller by about 4-fold. 

## 5. Conclusions

In summary, the mixture of the two non-ionic surfactants (Vit E-TPGS and OPEE) seems to be promising for the production of small nanomicelles as new system for the topical ocular delivery of Cyclosporine-A to treat dry eye syndrome. Their activity was remarkably improved by the combination of the nano-system with sodium hyaluronate. The main advantage of our delivery system consists in the remarkable increase in the residence time in tear fluid with a t1/2 value four times greater than that obtained with Ikervis, although a substantial modification of the pharmacokinetic profile could not be expected, as it is a aqueous colloidal dispersion. Another important advantage is represented by the technological characteristics of our formulation Nano1HAB-CyA: a transparent nano-colloidal dispersion compared to an opaque-white nanoemulsion (Ikervis), resulting in an improved chemical-physical stability and acceptability in patients.

Clinical studies will be necessary to identify the effective dose in the treatment of ocular pathologies such as DES, even if presumably the enhancement in bioavailability suggests a greater effectiveness. If a greater efficacy was also demonstrated in humans, a reduction of the instilled dose could be taken into account with the relative advantages.

## Figures and Tables

**Table 1:** Composition of the mixtures utilised for the preparation of ASMP-Nano. The formulations are identified with the following abbreviation: NanoXHA_y_-CyA, where X is total surfactant amount and y corresponds to the amount of hyaluronic acid.

N.	Formulations	Total surfactants (% *w*/*w*)	HA (%*w*/*w*)	Added CyA (% *w*/*w*)
1	Nano1.5HA_A_-CyA	1.50	0.001	0.15
2	Nano1.5HA_B_-CyA	1.50	0.01	0.15
3	Nano1.5HA_C_-CyA	1.50	0.10	0.15
4	Nano1HA_A_-CyA	1.00	0.001	0.15
5	Nano1HA_B_-CyA	1.00	0.01	0.15
6	Nano1HA_C_-CyA	1.00	0.10	0.15
7	Nano0.5HA_A_-CyA	0.50	0.001	0.15
8	Nano0.5HA_B_-CyA	0.50	0.01	0.15
9	Nano0.5HA_C_-CyA	0.50	0.10	0.15
10	Nano1-CyA (reference)	1.00	-	0.15
11	Nano1HA_B_ (reference)	1.00	0.01	-

**Table 2:** Analytical parameters for the different CyA calibration curves.

Medium	r^2^	LOQ^1^, μg/mL	LOD^2^, μg/mL
Acetonitrile	0.9979	0.030	0.010
Water	0.9992	0.560	0.170
Krebs Buffer Ringer	0.9997	0.050	0.015

^1^ Limit of Quantitation. ^2^ Limit of Detection.

**Table 3:** Experimental results of Design of Experiment (DOE) analysis for CyA content (CyA-In), hydrodynamic diameter (D_h_) and critical micellar concentration (CMC). The formulations are identified with the following abbreviation: NanoXHA_y_-CyA, where X is total surfactant amount and y corresponds to the amount of HA (A: 0.0001; B: 0.01; C: 0.1%).

Formulations	CyA-In (mean ± S.E. *n* = 3, % *w*/*w*)	D_h_ (mean ± S.D., *n* = 3, nm)	CMC (mean ± S.E. *n* = 3, % *w*/*w*)
Nano1.5 HA_A_-CyA	0.096 ± 0.002	14.15 ± 1.20	0.053 ± 0.007
Nano 1.5 HA_B_-CyA	0.098 ± 0.001	12.58 ± 1.60	0.059 ± 0.001
Nano 1.5 HA_C_-CyA	0.091 ± 0.003	13.75 ± 1.90	0.095 ± 0.005
Nano 1 HA_A_-CyA	0.087 ± 0.004	14.00 ± 3.20	0.042 ± 0.002
Nano 1 HA_B_-CyA	0.105 ± 0.003	14.41 ± 0.41	0.045 ± 0.003
Nano 1 HA_C_-CyA	0.082 ± 0.005	22.95 ± 5.60	0.057 ± 0.005
Nano 0.5 HA_A_-CyA	0.054 ± 0.002	13.07 ± 2.90	0.039 ± 0.005
Nano 0.5 HA_B_-CyA	0.046 ± 0.001	16.70 ± 5.20	0.044 ± 0.001
Nano 0.5 HA_C_-CyA	0.041 ± 0.002	12.25 ± 1.30	0.051 ± 0.004

**Table 4:** Main technological parameters for the selected formulations. Hydrodynamic Diameter (Dh), amount of solubilised CyA (CyA-In), nanomicelles Yield (ASMP-Y), CyA Entrapment Efficiency (CyA-EE) and CyA Loading Efficiency (CyA-LE).

	Nano1HA_B_ (n = 6, Mean ± SE)	Nano1HA_B_-CyA (n = 6, Mean ± SE)
Osmolality (mOsm/kg)	316 ± 0.33	316 ± 1.03
pH	6.8 ± 0.04	6.8 ± 0.05
D_h_	12.27 ± 1.29 ^§^	14.41 ± 0.41^§^
CyA-In (% *w*/*w*)	-	0.105 ± 0.003
ASMP-Y (% *w*/*w*)	-	95.74 ± 0.23
CyA-EE (% *w*/*w*)	-	77.66 ± 1.77
CyA-LD (% *w*/*w*)	-	10.40 ± 0.49

^§^ Standard deviation (*n* = 6).

**Table 5:** In vivo pharmacokinetics parameters of CyA in rabbit tear fluid (mean ± S.E., *n* = 6).

Formulation	C_1min_ (μg mL^−1^)	AUC (μg mL^−1^ min)	K_e_ (min^−1^)	t_1/2_ (min)
Nano1HA_B_-CyA	508.99 ± 45.47	2142 ± 233.6*	0.055± 0.009^#^	15.29 ± 3.49^#^
Nano1-CyA	458.18 ± 115.87	1426 ± 92.99	0.119 ± 0.038	7.12 ± 1.42
Ikervis	769.16 ± 43.50	1813 ± 354.1	0.205 ± 0.033	3.83 ± 0.74

^*^ significantly different from Nano1-CyA; ^#^significantly different from Ikervis®
